# Growth Hormone‐Related Gene Signatures Reveal Diagnostic Biomarkers, Immune Landscapes, and Potential Therapeutic Targets in Sepsis Through WGCNA and Machine Learning Analyses

**DOI:** 10.1002/iid3.70566

**Published:** 2026-10-28

**Authors:** Xiuhua Li, Ang Liu, Yingwei Ding, Gang Chen, Bingwen Zhang

**Affiliations:** ^1^ Department of Emergency Medicine, Affiliated Jinhua Hospital, Zhejiang University School of Medicine Jinhua Zhejiang China

**Keywords:** growth hormone‐related genes, immune infiltration, machine learning, sepsis, transcriptomic analysis

## Abstract

**Background:**

Sepsis reshapes inflammatory, immune, and metabolic activity, yet the behavior of genes linked to growth hormone biology within this response has not been well defined. We therefore searched for growth hormone‐related genes (GHRGs) that could distinguish sepsis and asked whether the resulting markers also reflected variation in host immune state.

**Methods:**

For model development, two Gene Expression Omnibus (GEO) microarray cohorts were combined, while GSE26378 and GSE28750 were kept separate for validation. Sepsis‐associated expression signals were first defined by differential analysis and weighted gene co‐expression network analysis (WGCNA), then intersected with 1205 GHRGs. A protein–protein interaction step and four machine‐learning procedures were used to reduce the candidate set. Classification was examined with receiver operating characteristic (ROC) curves and a two‐gene nomogram, with calibration and decision‐curve analyses used as complementary checks. We also examined pathway enrichment, immune‐cell patterns, predicted regulatory interactions, candidate compounds, and expression‐based molecular subgroups.

**Results:**

The integrated cohort yielded 2552 DEGs and 1873 genes from sepsis‐associated WGCNA modules; their overlap with the GHRG collection contained 40 genes. PPI module analysis reduced this set to 23 hub candidates. Machine‐learning screening consistently retained JAK1 and GATA3, both of which were downregulated in sepsis and showed stable diagnostic performance across the training and external validation cohorts. Their combined model provided stronger discrimination than either gene alone. Functional analyses linked the two genes to T‐cell signaling, antigen processing, immune regulation, mitochondrial metabolism, and translational processes. Immune profiling revealed reduced lymphocyte‐associated features together with enhanced myeloid signatures in sepsis. When septic samples were grouped by JAK1/GATA3 expression, two subtypes with different immune and transcriptional profiles emerged.

**Conclusions:**

JAK1 and GATA3 emerged as promising growth hormone‐related candidate diagnostic biomarkers associated with immune heterogeneity in sepsis. Their combined signature may provide a useful framework for molecular stratification and warrants further experimental and clinical validation.

## Highlights

- Integrative transcriptomic and machine‐learning analyses identified JAK1 and GATA3 as growth hormone‐related diagnostic biomarkers with stable performance across independent sepsis cohorts.
- JAK1 and GATA3 were closely associated with T‐cell signaling, antigen processing, immune‐cell redistribution, and broader immune dysregulation in sepsis.
- JAK1/GATA3‐based consensus clustering defined two molecular subtypes of sepsis with distinct transcriptional programs and immune infiltration landscapes.

## 1 Introduction

Sepsis is defined as organ dysfunction caused by a dysregulated host response to infection [1], but the biological response behind that definition is far from uniform. Inflammatory and counter‐regulatory programs may be active at the same time, while antigen presentation, leukocyte abundance, lymphocyte function, cellular metabolism, and tissue homeostasis all change as illness evolves [2]. In particular, lymphocyte apoptosis and dysfunction, reduced antigen‐presenting capacity, altered co‐stimulatory signaling, T‐cell exhaustion, and expansion of immunoregulatory or myeloid populations can produce sustained immune suppression in a subset of patients [3]. This variation in host response is one reason that clinically similar patients can diverge substantially in subsequent course. Accordingly, molecular markers that distinguish sepsis while also capturing host immune state may provide information that is not fully represented by conventional clinical assessment alone. Transcriptomic studies have already shown that host–response signatures can discriminate infectious states and reveal reproducible sepsis endotypes, supporting the feasibility of gene‐expression‐based patient classification [4].

Endocrine and metabolic adaptation is closely intertwined with the immune response during critical illness, making growth hormone (GH)‐related biology a potentially informative but insufficiently explored context for sepsis. Canonical signaling through the GH receptor is centered on JAK2 and downstream STAT, PI3K, ERK, and related pathways; it is mechanistically distinct from JAK1‐dependent signaling used by many cytokine receptors [5]. During experimental endotoxemia, inflammatory stress can produce postreceptor GH resistance, with reduced GH‐stimulated JAK2/STAT5 activity and increased suppressors of cytokine signaling [6]. GH can also influence immune‐cell cytokine production, further supporting bidirectional interactions between endocrine and inflammatory systems [7]. These observations provide a biological rationale for examining genes associated with GH‐related processes in sepsis, but they do not imply that altered GH signaling is a direct cause of the disease. Indeed, the complexity of this relationship is underscored by randomized evidence showing increased mortality after high‐dose GH administration in adults with prolonged critical illness [8]. Thus, a transcriptomic “growth hormone‐related” framework is more appropriately viewed as a means of interrogating endocrine–immune‐associated molecular programs than as evidence supporting GH supplementation.

Despite increasing interest in transcriptome‐based biomarkers for sepsis, several issues remain unresolved. Many previous studies have focused on genome‐wide differential expression or predefined immune‐related gene sets, whereas endocrine–immune‐associated transcriptional programs have received less systematic attention [9, 10]. Recent work has integrated NET‐ and inflammation‐related signatures with immune deconvolution to model sepsis‐induced acute lung injury [11]. Moreover, candidate biomarkers derived from a single analytical strategy may be sensitive to cohort composition and feature‐selection bias. Integrating disease‐associated co‐expression networks with multiple machine‐learning approaches may therefore provide a more stringent framework for identifying reproducible molecular features. More importantly, diagnostic discrimination alone does not capture the marked biological heterogeneity of sepsis. Linking candidate biomarkers to immune‐cell composition and functional states may help clarify whether they also reflect clinically relevant differences in host‐response patterns.

The present study aimed to characterize the diagnostic and immunological significance of growth hormone‐related transcriptional alterations in sepsis. By integrating multiple independent transcriptomic cohorts with co‐expression network and machine‐learning approaches, we sought to identify robust GH‐related molecular features associated with sepsis and to determine whether these features reflect underlying differences in host immune status. We further explored their functional context and their potential to distinguish biologically distinct patient subgroups. Collectively, this study was designed to provide a transcriptomic framework for understanding the relationship between GH‐related molecular programs, diagnostic signatures, and immune heterogeneity in sepsis.

## 2 Methods

### 2.1 Data Acquisition and Gene Set Curation

The discovery analysis used two public Gene Expression Omnibus (GEO, https://www.ncbi.nlm.nih.gov/geo/) microarray datasets. GSE57065 contributed 107 samples (82 sepsis and 25 non‐septic controls), and GSE95233 contributed 124 samples (102 sepsis and 22 controls). After the expression matrices were combined, dataset‐related batch structure was adjusted with limma::removeBatchEffect, leaving a training set of 231 samples: 184 sepsis cases and 47 controls. Two additional datasets were not included in model fitting. GSE26378 contained 82 sepsis and 21 control samples, and GSE28750 contained 10 sepsis and 20 controls; both were used only for external validation. Notably, GSE28750 originally contained 10 sepsis samples, 11 post‐operative sepsis samples, and 20 control samples. To maintain phenotypic consistency across samples, only the 10 sepsis and 20 control samples were retained for analysis. Detailed sample information for all datasets is provided in Supporting Information S2: Table S1. Because GSE57065 and GSE95233 contain longitudinal samples collected from the same patients at different time points, we calculated pairwise gene expression correlations among samples using the top 2000 highly variable genes to assess potential within‐patient dependence. Growth hormone‐associated genes were retrieved from GeneCards (https://www.genecards.org/) on July 25, 2026, by searching “Growth hormone.” Applying a relevance‐score cutoff greater than 15 produced 1205 GHRGs, which are listed in Supporting Information S2: Table S2.

### 2.2 Identification of Differentially Expressed Genes (DEGs)

Within the merged training matrix, septic‐versus‐control expression contrasts were estimated with limma. Gene‐wise linear models were moderated by empirical Bayes shrinkage, and Benjamini–Hochberg adjustment was used for multiple testing. We classified a transcript as differentially expressed when the adjusted *P* value was below 0.05, and the absolute log2 fold change exceeded 0.585.

### 2.3 Weighted Gene Co‐Expression Network Analysis (WGCNA)

Co‐expression structure in the training cohort was modeled with WGCNA. Sample clustering was inspected first so that gross outliers could be identified before network fitting. Network construction used the 5000 genes with the largest across‐sample variability. A power of *β *= 6 gave a scale‐free topology fit of *R* = 0.85 and was therefore used to transform correlations. Dynamic tree cutting, with 50 genes as the minimum module size, defined the co‐expression modules. Module eigengenes were then related to sepsis status by Pearson correlation. Modules showing significant relationships with disease status (correlation coefficient > 0.3 and *p* < 0.05) were considered phenotype‐associated modules, and genes contained within these modules were retained as WGCNA‐derived candidate genes.

### 2.4 Identification of Differentially Expressed GHRGs (DEGHRGs)

The disease‐associated list was refined by requiring support from three sources: differential expression, membership in a sepsis‐associated WGCNA module, and inclusion in the GeneCards‐derived GHRG set. Genes present in all three lists were termed DEGHRGs and were taken into the interaction‐network and machine‐learning stages.

### 2.5 Network‐Topology Filtering of DEGHRGs

STRING (https://string-db.org/) was queried with the DEGHRG set, retaining edges with a minimum confidence score greater than 0.15. The resulting interaction graph was transferred to Cytoscape 3.10.4 (https://cytoscape.org/) for visualization and topology‐based module screening. MCODE was run with degree cutoff = 2, node score cutoff = 0.2, K‐core = 2, and maximum depth = 100. Rather than treating every network node equally, we selected the module with the highest MCODE score and defined its member genes as candidate hubs. Expression patterns of hub genes between septic and control samples were further evaluated in the training cohort for subsequent diagnostic assessment.

### 2.6 Machine Learning‐Based Feature Selection

Feature reduction was repeated under four algorithms with different inductive biases: least absolute shrinkage and selection operator (LASSO), extreme gradient boosting (XGBoost), support vector machine‐recursive feature elimination (SVM‐RFE), and random forest (RF). To ensure reproducibility, a fixed random seed (set. seed(123)) was applied before model training. LASSO regression was performed using the glmnet package. Ten‐fold cross‐validation was used to determine the optimal regularization parameter (lambda), and key genes were selected according to the minimum error criterion (lambda.min). XGBoost was implemented using the xgboost package, with the main parameters set as max_depth = 5, eta = 1, nrounds = 100, and objective = “binary:logistic.” Genes with feature importance greater than 0 were retained as candidate features. Random forest (RF) was constructed using the randomForest package (ntree = 5000). Variables were ranked by mean decrease in the Gini index (MeanDecreaseGini), and genes with MeanDecreaseGini > 2 were included in subsequent analyses. Support vector machine–recursive feature elimination (SVM‐RFE) was performed using the e1071 package. The parameter halve.above = 100 was set to ensure that features were eliminated one by one once the number of remaining features fell below this threshold. The optimal feature combination was determined through 10‐fold cross‐validation (*k *= 10) by identifying the gene set with the lowest cross‐validation error rate. The intersection of the genes selected by these four methods yielded feature genes with potential diagnostic value.

To avoid potential bias introduced by repeated samples from the same patient in 10‐fold cross‐validation, all samples derived from the same patient were assigned to the same fold during data splitting. The machine learning analyses were then repeated under this patient‐level cross‐validation scheme to identify robust feature genes.

### 2.7 Construction and Validation of the Diagnostic Model

Diagnostic assessment began in the merged discovery cohort. Receiver operating characteristic (ROC) curves were generated with pROC, and area under the curve (AUC) > 0.70 was used as the threshold for retaining an individual marker. The two final genes were then entered together into a diagnostic model that was represented as a nomogram. Agreement between fitted and observed probabilities was examined by calibration, and RMDA‐based decision‐curve analysis was used to estimate net benefit over a range of thresholds. All model fitting was confined to the discovery data. GSE26378 and GSE28750 were evaluated afterward as separate external cohorts, using ROC analysis and gene‐expression comparisons without refitting the discovery procedure.

### 2.8 Functional Enrichment Analysis

Enrichment analyses were used at several stages to interpret gene sets rather than to select the final diagnostic markers. WGCNA‐derived genes, PPI hub genes, and subtype‐associated DEGs were analyzed with clusterProfiler. Gene Ontology (GO) biological‐process terms and Kyoto Encyclopedia of Genes and Genomes (KEGG) pathways were tested with clusterProfiler in R, and statistically supported annotations were retained under the predefined enrichment criteria.

### 2.9 Single‐Gene Enrichment Analysis

For gene‐centered functional analysis, separate ranked lists were created for JAK1 and GATA3. The reference collections c5.go.bp.v2026.1.Hs.symbols and c2.cp. kegg_medicus.v2026.1.Hs.symbols were downloaded from MSigDB (https://www.gsea-msigdb.org/gsea/index.jsp). Across the transcriptome, genes were ordered by their expression correlation with the marker under study. Gene set enrichment analysis (GSEA) was then applied to each ranked list, allowing GO biological processes and KEGG Medicus pathways associated with JAK1‐ or GATA3‐linked expression patterns to be identified.

### 2.10 Immune Infiltration Analysis

Immune variation was examined from three complementary perspectives. First, single‐sample gene set enrichment analysis (ssGSEA) converted each transcriptome into scores for immune‐cell signatures and immune‐related functions, which were compared between sepsis and control samples. Second, CIBERSORT was used to deconvolute relative leukocyte fractions. Third, MCP‐counter within IOBR provided abundance estimates for additional immune and stromal populations. To connect these profiles with the diagnostic markers, Pearson coefficients were calculated between JAK1 or GATA3 expression and the corresponding immune estimates.

### 2.11 Integration of Upstream‐Regulator Evidence and Compound Associations

Putative transcriptional regulators of JAK1 and GATA3 were collected from NetworkAnalyst (https://www.networkanalyst.ca/), whereas miRNet (https://www.mirnet.ca/) supplied predicted miRNA‐gene links. The two relationship sets were assembled and displayed in Cytoscape 3.10.4. Regulatory nodes were ranked with cytoHubba using Maximum Clique Centrality, and the 20 highest ranked nodes were retained for presentation. For compound prioritization, the two diagnostic genes were queried against DSigDB (http://dsigdb.tanlab.org/DSigDBv1.0/) through the Enrichr interface (https://maayanlab.cloud/modEnrichr/). Enriched drug‐gene associations were treated as candidate compounds linked to JAK1 and/or GATA3.

### 2.12 Molecular Subtype Identification

Molecular subtyping was performed exclusively on sepsis samples in the training cohort. After extracting the expression values of the two hub genes, JAK1 and GATA3, from the normalized expression matrix, consensus clustering was carried out using the ConsensusClusterPlus R package. The analysis was based on 100 resampling iterations, with 80% of samples resampled in each iteration (pItem = 0.8) and all features retained (pFeature = 1). K‐means clustering with Euclidean distance was used as the underlying clustering algorithm, and the number of clusters was evaluated from *k* = 2 to 9. A fixed random seed (seed = 123456) was set to ensure reproducibility. The optimal number of clusters was determined by calculating the proportion of ambiguous clustering (PAC) for each *k*, with the *k* value yielding the lowest PAC selected as the optimal solution. Principal component analysis (PCA) was subsequently performed as an independent visual assessment of subgroup separation. Marker expression across the resulting subtypes was summarized with boxplots. The immune comparison reused the ssGSEA, CIBERSORT, and MCP‐counter procedures described in Section 2.10. Subtype‐to‐subtype differential expression was defined by adjusted *p* < 0.05 together with |log_2_FC | > 0.585. Those DEGs were displayed in a volcano plot and interpreted with the enrichment workflow described in Section 2.8.

### 2.13 Experimental HUVEC Response to Lipopolysaccharide (LPS) Exposure

Human umbilical vein endothelial cells (HUVECs; #iCell‐h110, iCell Biotech, Shanghai, China) provided the experimental system for checking JAK1 and GATA3 expression. Cells were propagated in the manufacturer's HUVEC medium (#iCell‐h110‐001b, iCell Biotech) and used between three and six passages. For inflammatory stimulation, cultures received 300 ng/mL LPS (#L8880, Solarbio, Beijing, China) for 24 h; parallel cultures receiving no LPS served as controls. RNA was collected from both conditions at the end of the exposure period and used for downstream reverse transcription reverse transcription quantitative real‐time PCR (RT‐qPCR). The experiments were performed in four independent biological replicates, with each biological replicate measured in three technical replicates.

### 2.14 RT‐qPCR

RNA from untreated and LPS‐exposed HUVECs was isolated with TRIzol (#15596026CN, Invitrogen, Gaithersburg, MD, USA). cDNA was generated with the First Strand cDNA Synthesis Kit (#G3333‐50, Servicebio, Wuhan, China). Quantification was carried out on a QuantStudio 1 Real‐Time PCR System (Thermo Fisher Scientific, Waltham, MA, USA) with 2× SYBR Green qPCR Master Mix (Servicebio). GAPDH served as the reference transcript for JAK1 and GATA3, and relative abundance was expressed by the 2^−ΔΔCt^ method using the untreated condition as the calibrator. Primer sequences are listed in Table 1. Group comparisons were performed using unpaired two‐tailed *t*‐test. Data are presented as mean ± SD. A two‐sided *p* < 0.05 was considered statistically significant.

**Table 1:** Sequences of primers for qRT‐PCR.

Gene name	Primer sequences (5′−3′)
JAK1	Forward: GCATCGAGCGCACAAAGTTAT
	Reverse: CAGGCACCAGTAGGGTTGAG
GATA3	Forward: ACCACCCCATCACCACCTA
	Reverse: CACACTCCCTGCCTTCTGTG
GAPDH	Forward: GCTCTCTGCTCCTCCTGTTC
	Reverse: ACGACCAAATCCGTTGACTC

### 2.15 Statistical Analysis

Analyses were run in R 4.4.1 with the packages specified above. Two independent groups were compared with the Wilcoxon rank‐sum test where applicable. ROC calculations used pROC, and most plots were drawn with ggplot2. Pearson correlation was used for continuous‐variable associations. Unless a section states otherwise, tests were two‐sided and *p* < 0.05 defined statistical significance. Figure annotations used **** for *p* < 0.0001, *** for *p* < 0.001, ** for *p* < 0.01, * for *p* < 0.05, and ns for *p* ≥ 0.05.

## 3 Results

### 3.1 Expression Changes After Harmonizing the Two Discovery Cohorts

To establish a reliable training cohort, transcriptomic profiles from GSE57065 and GSE95233 were integrated, followed by batch effect adjustment. PCA largely separated samples by dataset source, consistent with a strong technical component (Figure 1A). After batch adjustment, samples from GSE57065 and GSE95233 showed improved integration and reduced dataset‐specific clustering patterns, suggesting that the major technical bias had been effectively alleviated (Figure 1B). Because GSE57065 and GSE95233 contain longitudinal samples sequenced from the same patients at different time points, we calculated pairwise sample correlations based on the expression of the top 2000 highly variable genes. We found that samples from the same patient did not always show the highest correlation (Supporting Information S1: Figure S1A, S1B). Subsequently, differential expression profiling was conducted between septic and control samples in the integrated cohort. A total of 2552 DEGs were identified according to the predefined criteria, including 1239 upregulated genes and 1313 downregulated genes (Figure 1C).

**Figure 1:**
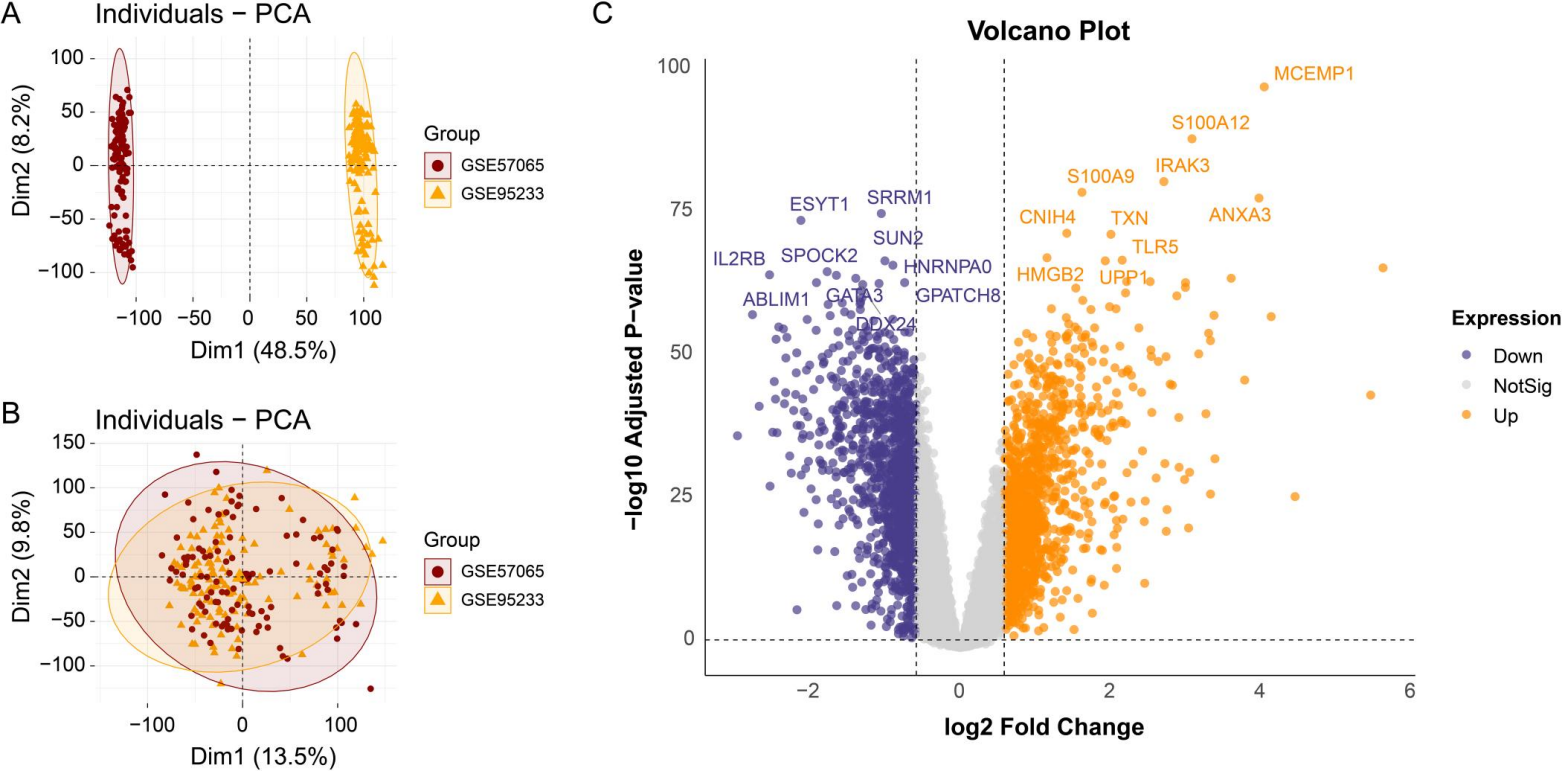
Integration of GEO cohorts and identification of DEGs in sepsis. (A) PCA before removal of dataset‐origin effects, showing the relative positions of GSE57065 and GSE95233 samples. (B) PCA of the same merged cohort after batch adjustment. (C) Volcano representation of sepsis‐control expression differences, with genes passing the DEG criteria distinguished from nonsignificant genes.

### 3.2 Identification of Sepsis‐Associated Co‐Expression Modules and Growth Hormone‐Related Candidate Genes

At β = 6, the WGCNA network satisfied the selected scale‐free topology criterion (Figure 2A). Dynamic tree cutting then partitioned the transcriptome into multiple modules (Figure 2B). Four module eigengenes were positively related to sepsis status: blue (*r* = 0.88), black (*r* = 0.38), green (*r *= 0.36), and salmon (*r* = 0.31) (Figure 2C). Together, they contained 1873 genes. Functional annotation placed many of these genes in adaptive‐immune and lymphocyte programs, including antigen‐receptor signaling, immune‐response‐regulating cell‐surface receptor signaling, T‐cell differentiation, and lymphocyte differentiation. Ribosomal subunits, rRNA binding, immune‐receptor activity, and structural constituents of ribosomes were also represented (Figure 2D). KEGG results emphasized T‐cell receptor signaling, antigen processing and presentation, hematopoietic cell lineage, Th17 differentiation, and Th1/Th2 differentiation, with additional signals for PD‐L1/PD‐1 checkpoint biology and primary immunodeficiency (Figure 2E). Thus, the sepsis‐associated modules were dominated by immune programs rather than a single isolated pathway. Intersecting their 1873 genes with the 2552 DEGs and 1205 GHRGs left 40 shared DEGHRGs (Figure 2F).

**Figure 2:**
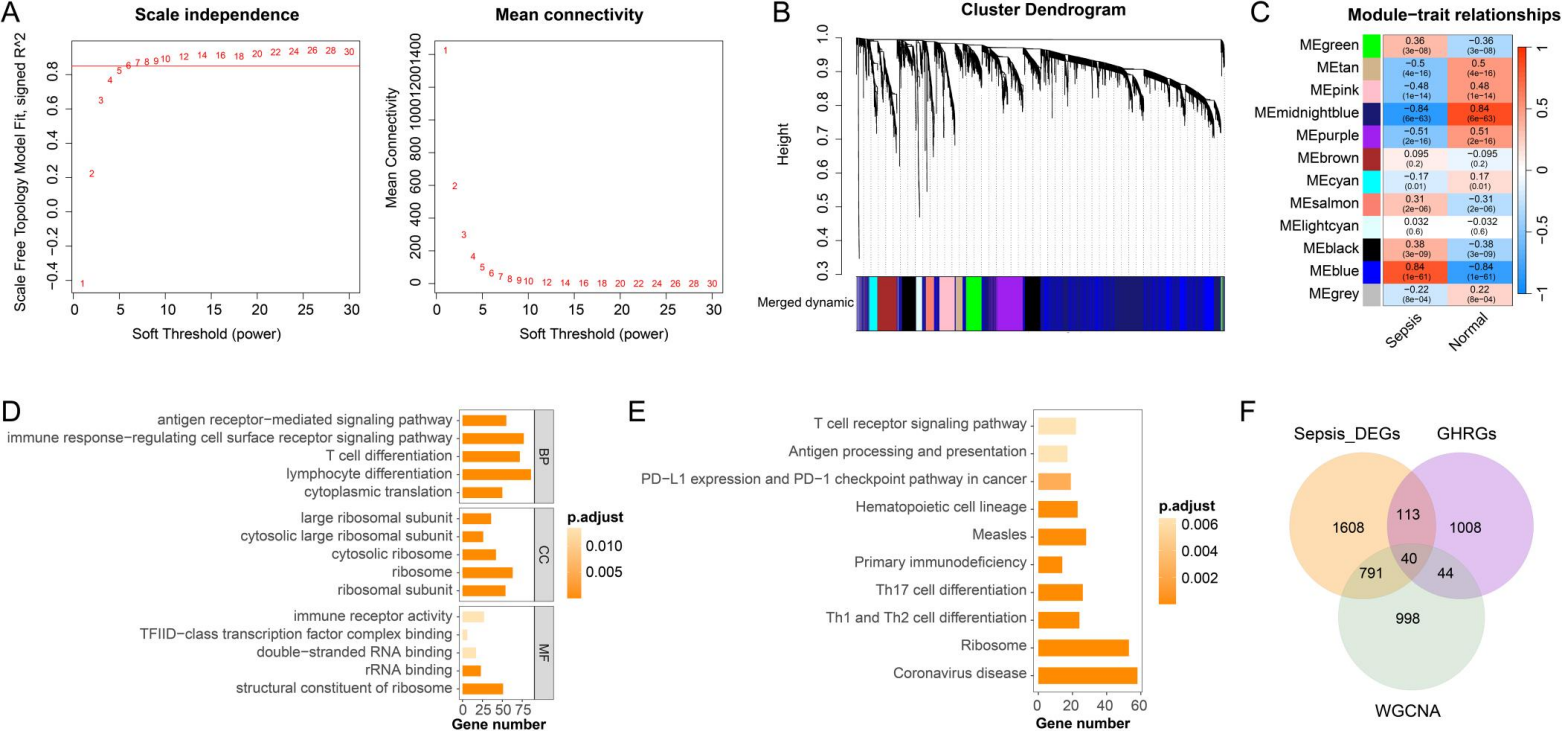
WGCNA‐based definition of sepsis‐associated modules and their overlap with GHRGs. (A) Scale‐independence and mean‐connectivity curves used to select the soft‐thresholding power. (B) Module assignment along the hierarchical gene dendrogram. (C) Heatmap relating module eigengenes to sepsis/control status. (D) GO terms enriched among genes from phenotype‐associated modules. (E) KEGG pathways represented by the same module genes. (F) Three‐way overlap of DEGs, WGCNA‐derived genes, and the GeneCards GHRG set.

### 3.3 Extraction of a 23‐Gene Interaction Hub From the DEGHRG Network

The 40 DEGHRGs formed a connected STRING network for many of the included proteins (Figure 3A). Module screening with MCODE further identified one densely interconnected subnetwork comprising 23 genes, which were retained as candidate hub genes for subsequent analyses (Figure 3B). Expression profiling showed clear differences in the abundance of multiple hub genes between septic and control samples (Figure 3C), further supporting their potential relevance to the disease‐associated transcriptional landscape. GO annotation linked the 23 hubs to hematopoietic regulation, alpha‐beta T‐cell differentiation, myeloid differentiation, intrinsic apoptotic signaling, transcriptional control, and protein binding (Figure 3D). KEGG terms included JAK‐STAT signaling, Th1/Th2 differentiation, Th17 differentiation, and apoptosis; pathways annotated to several viral or parasitic infections were also represented (Figure 3E). Taken together, the functional profiles of the PPI‐derived hub genes pointed to prominent involvement in immune regulation, hematopoietic processes, and cell‐death‐associated signaling in sepsis.

**Figure 3:**
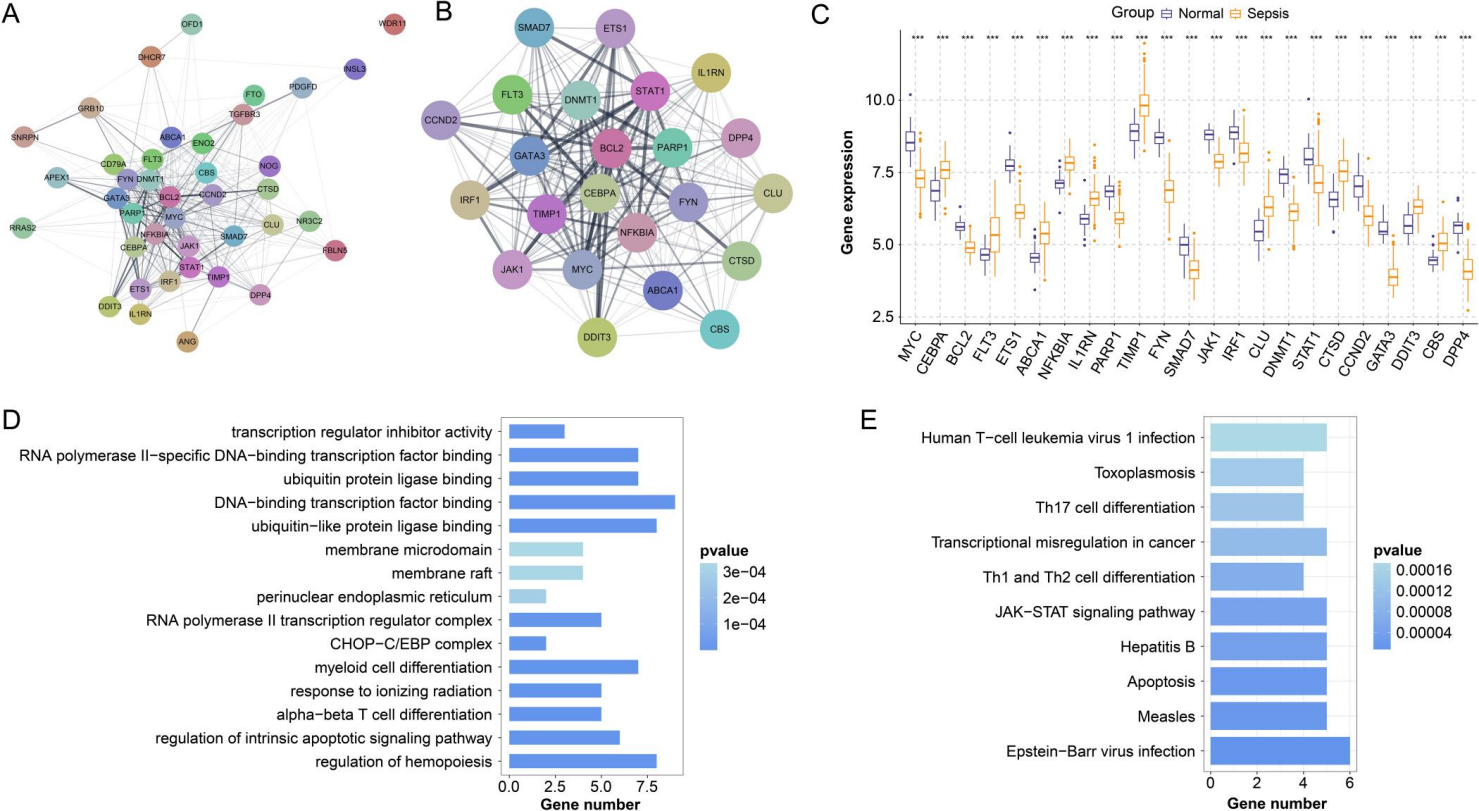
Network‐based refinement and functional annotation of DEGHRGs. (A) PPI network constructed from the 40 DEGHRGs. (B) Highest‐scoring MCODE subnetwork, containing 23 candidate hubs. (C) Training‐cohort expression patterns for these hub genes in sepsis and control samples. (D) GO enrichment analysis of the PPI‐derived hub genes. (E) KEGG annotations associated with the same hub set.

### 3.4 Machine Learning‐Based Identification of Diagnostic Genes

The 23 hub genes were next challenged under four independent feature‐selection procedures. LASSO retained 10 genes at the cross‐validated penalty setting (Figure 4A,B). XGBoost ranked the contribution of individual genes to classification and selected five features with positive gain values, among which GATA3 showed the highest feature importance, followed by FYN, ETS1, PARP1, and JAK1 (Figure 4C). Under 10‐fold cross‐validation, the lowest SVM‐RFE error corresponded to an eight‐gene feature set (Figure 4D). Random‐forest error approached a plateau as tree number increased (Figure 4E). Variable‐importance assessment based on MeanDecreaseGini retained 11 genes according to the predefined threshold, with GATA3, FYN, and ETS1 ranking among the most informative features (Figure 4F). Thus, LASSO, XGBoost, SVM‐RFE, and random forest selected 10, 5, 8, and 11 candidate genes, respectively; the complete gene lists derived from each algorithm are provided in Supporting Information S2: Table S3. Their intersection contained only JAK1 and GATA3, which were carried forward as the most reproducible candidates (Figure 4G). To mitigate potential bias from repeated samples of the same patient, we performed patient‐level 10‐fold cross‐validation by assigning all samples from the same patient to the same fold. Re‐running the machine learning analyses under this scheme again yielded JAK1 and GATA3 as the selected feature genes (Supporting Information S2: Figure S2A–S2G). Meanwhile, we collected 85 GH‐related genes from MSigDB and the literature, intersected them with differentially expressed genes and WGCNA hub genes, and applied machine learning algorithms to identify feature genes. This analysis yielded three feature genes, among which JAK1 and GATA3 were also identified (Supporting Information S2: Figure S3A–S3H).

**Figure 4:**
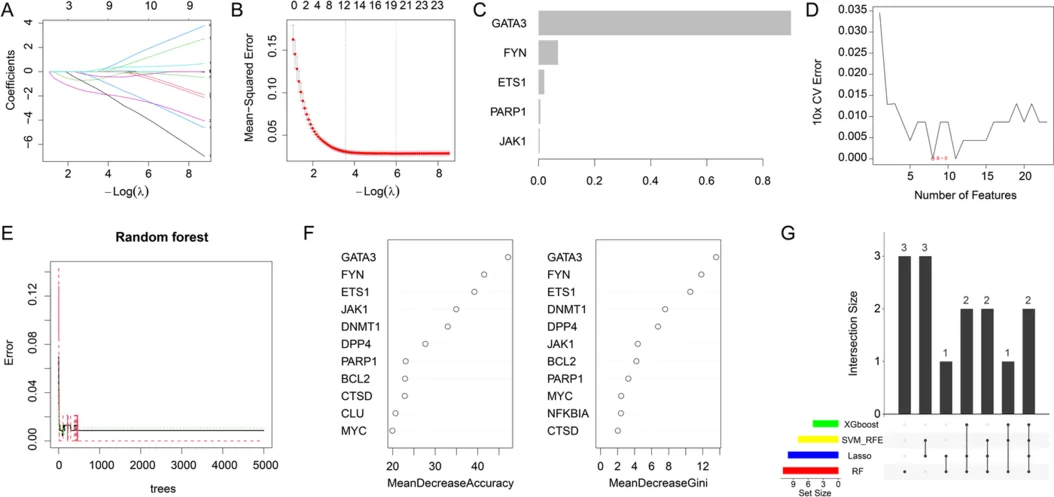
Machine learning‐based screening of candidate diagnostic genes. (A) LASSO coefficient trajectories as regularization changes. (B) Cross‐validation profile used to choose the LASSO penalty. (C) XGBoost gain‐based feature ranking. (D) SVM‐RFE cross‐validation error across candidate feature‐set sizes. (E) Random‐forest error as tree number increased. (F) Random‐forest importance metrics. (G) UpSet plot showing the intersection of the four selected gene lists.

### 3.5 Diagnostic Performance and Expression Patterns of JAK1 and Gata3

Both markers discriminated sepsis from controls in the training cohort with AUC values above 0.70, while the model containing JAK1 and GATA3 together performed best (Figure 5A). Both transcripts were lower in sepsis than in controls (Figure 5B). The same direction of expression change was retained in GSE26378 and GSE28750, where the combined classifier also remained informative (Figure 5C–F). A JAK1/GATA3 nomogram was then used to translate the fitted model into individual diagnostic probabilities (Figure 5G). Decision‐curve analysis showed positive net benefit across a broad threshold range (Figure 5H), and calibration indicated close correspondence between fitted and observed probabilities (Figure 5I). Furthermore, decision curve and calibration analyses conducted in the GSE26378 validation cohort demonstrated findings comparable to those in the discovery set (Supporting Information S1: Figure S4A, S4B).

**Figure 5:**
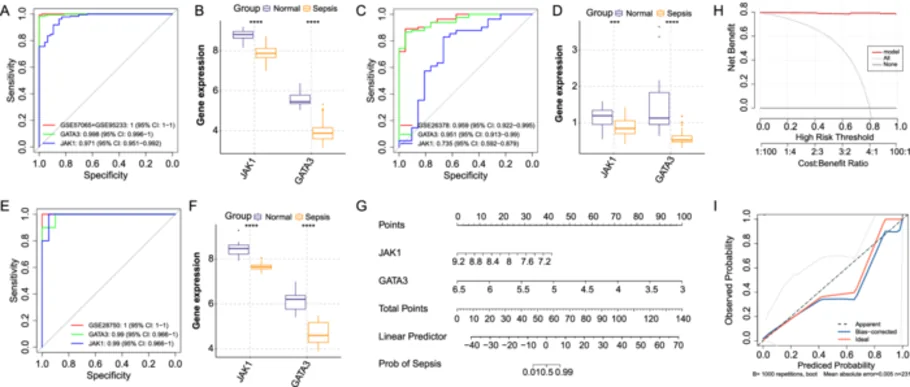
Diagnostic evaluation and validation of JAK1 and GATA3 in sepsis. (A) Training‐cohort ROC curves for JAK1, GATA3, and their combined model. (B) Training‐cohort expression distributions for the two genes. (C, D) ROC performance and gene‐expression distributions, respectively, in GSE26378. (E, F) Corresponding ROC and expression results in GSE28750. (G) Nomogram derived from the two markers. (H) Decision curve for the paired‐gene model. (I) Calibration of predicted sepsis probability against the observed outcome.

### 3.6 Single‐Gene Enrichment Analysis of JAK1 and GATA3

The ranked‐expression analyses placed JAK1 and GATA3 within overlapping, but not identical, functional contexts. For JAK1, GO‐BP terms toward one end of the ranked list included antigen‐receptor signaling, lymphocyte activation, T‐cell activation, and T‐cell receptor signaling; ATP‐synthesis‐coupled electron transport and electron‐chain signatures appeared in the opposite direction (Figure 6A). KEGG Medicus results similarly linked JAK1‐related variation to MHC class II antigen processing/presentation, mitochondrial complex I and electron transfer, and translation initiation (Figure 6B). These results indicated that JAK1 expression was linked not only to immune recognition and antigen presentation but also to cellular bioenergetic processes. GATA3‐associated GO terms were dominated by antigen‐receptor and T‐cell signaling together with ribonucleoprotein‐complex biogenesis, ribosome biogenesis, rRNA processing, and translation (Figure 6C). KEGG analysis similarly revealed enrichment of pathways involving MHC class II‐mediated antigen processing and presentation, T‐cell receptor‐related signaling, and translation initiation, alongside additional immune‐regulatory signaling events (Figure 6D). Taken together, the enrichment profiles of JAK1 and GATA3 converged on antigen‐mediated immune responses and T‐cell‐associated signaling, while also revealing distinct links to mitochondrial metabolism and translational regulation. These findings provided additional functional context for the potential involvement of both genes in the immune dysregulation characteristic of sepsis.

**Figure 6:**
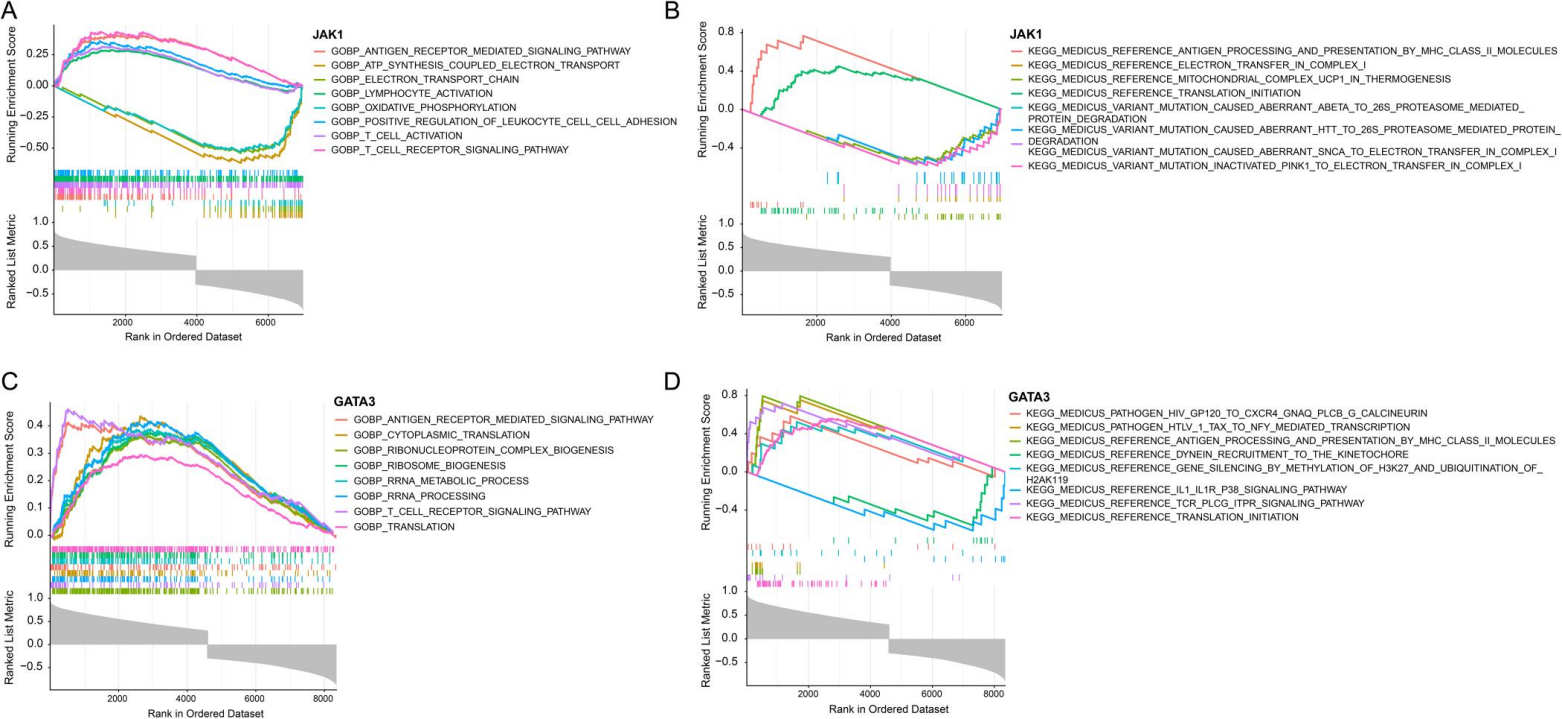
Single‐gene enrichment analysis of JAK1 and GATA3. (A) GO‐BP enrichment profiles associated with JAK1 expression. (B) KEGG Medicus pathway enrichment profiles associated with JAK1 expression. (C) GO‐BP enrichment profiles associated with GATA3 expression. (D) KEGG Medicus pathway enrichment profiles associated with GATA3 expression.

### 3.7 Sepsis‐Associated Immune Shifts Tracked by JAK1 and GATA3

ssGSEA showed that sepsis was accompanied by broad changes in inferred immune composition (Figure 7A). Scores for activated dendritic cells, B cells, CD8 + T cells, immature dendritic cells, mast cells, natural killer cells, plasmacytoid dendritic cells, T‐helper cells, T‐follicular helper cells, and several other T‐cell subsets were generally lower than in controls, whereas dendritic‐cell, Treg, macrophage, and neutrophil signatures tended to be higher. Functional scores were also redistributed (Figure 7B), affecting T‐cell co‐stimulation, checkpoint activity, cytolytic activity, HLA‐associated responses, inflammation‐promoting activity, and type I interferon responses. A second view from CIBERSORT identified group differences in plasma cells, CD8 + T cells, naive CD4 + T cells, resting NK cells, M0 macrophages, and neutrophils (Figure 7C), again suggesting lower lymphocyte representation with stronger myeloid signals in sepsis. MCP‐counter pointed in the same general direction: several T‐cell, cytotoxic, B‐lineage, NK‐cell, and monocytic‐lineage estimates differed between groups, while neutrophils were higher in sepsis; myeloid dendritic cells and fibroblasts were not significantly different (Figure 7D). JAK1 expression rose with many lymphocyte‐associated features, including TIL‐like signatures, T‐cell co‐stimulation, HLA activity, T helper cells, checkpoint scores, CD8 + T cells, cytolytic activity, and Th1‐cell signals, but decreased as macrophage‐related estimates increased (Figure 7E). GATA3 showed a similar pattern, with positive associations across T‐cell/cytotoxic features and a strong inverse association with macrophage signatures (Figure 7F). Overall, lower JAK1/GATA3 expression occurred in a transcriptomic environment with weaker lymphocyte‐associated activity and relatively stronger myeloid signals.

Figure 7Immune changes in sepsis and their relationship with the two diagnostic markers. (A, B) ssGSEA cell and immune‐function scores. (C) CIBERSORT fractions differing between sepsis and controls. (D) MCP‐counter abundance estimates. (E, F) Correlations of JAK1 and GATA3 with ssGSEA immune features.

**Figure:**
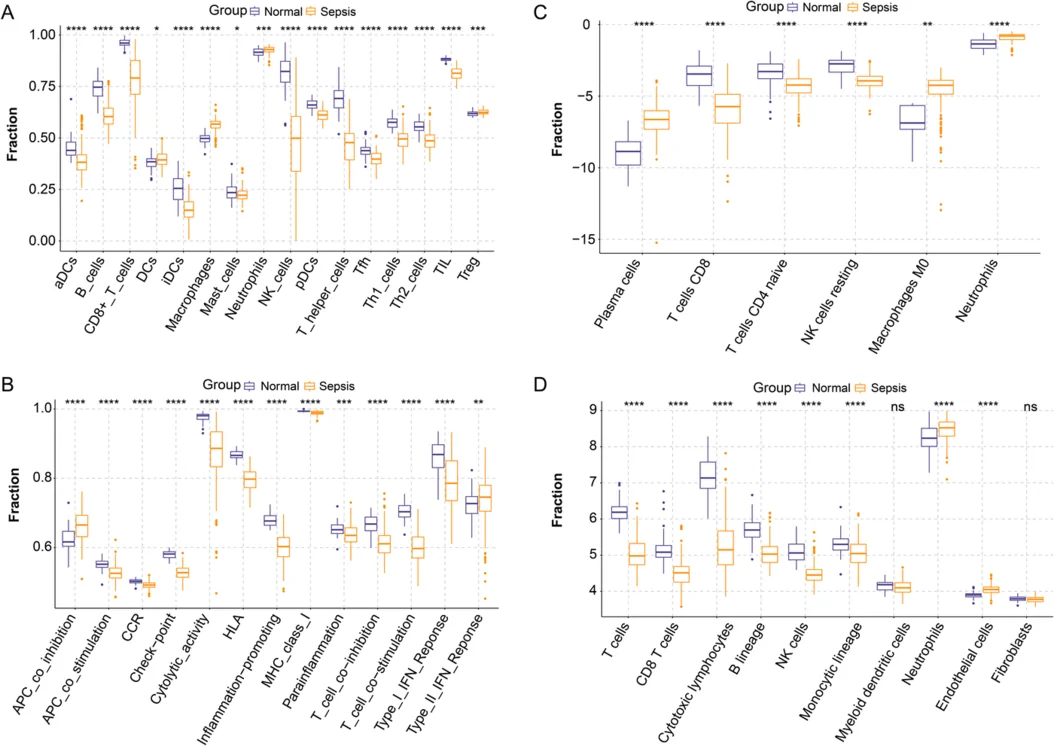

**Figure:**
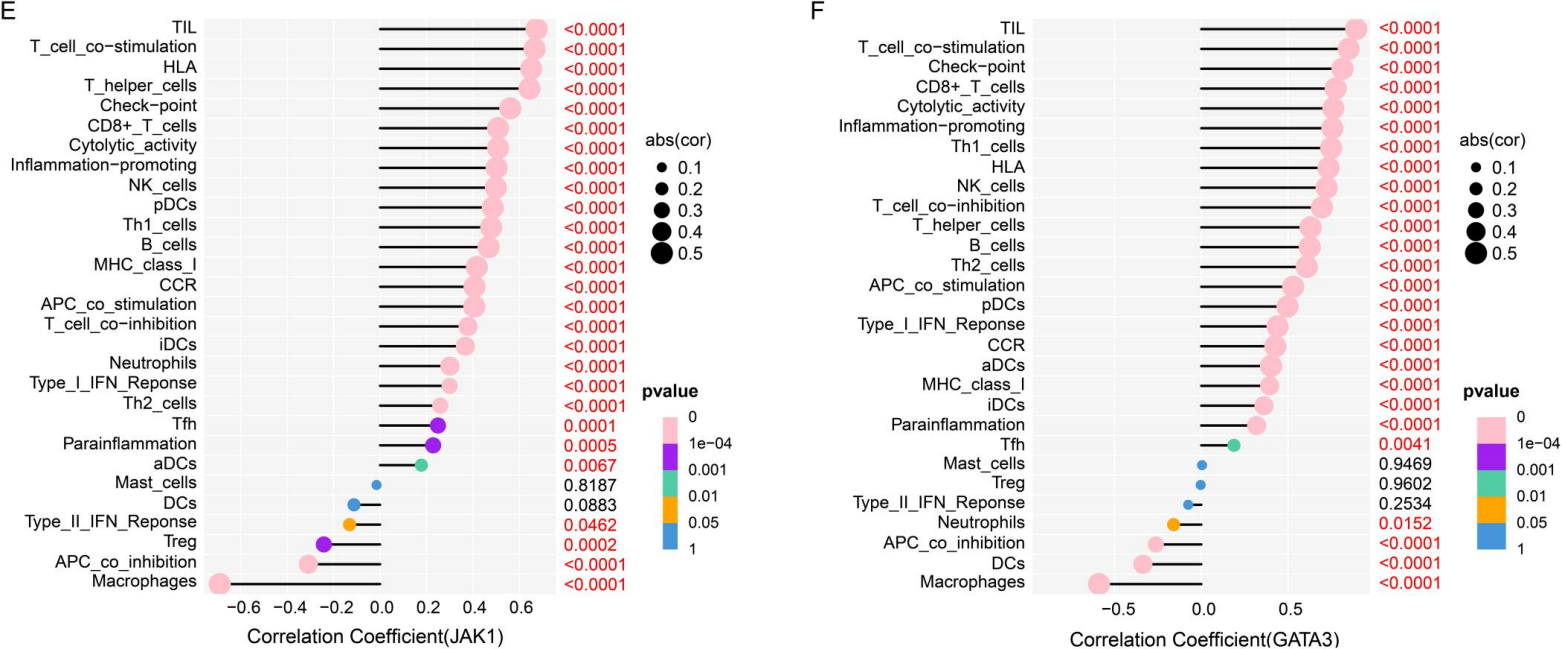

### 3.8 Candidate Regulators and Compound Associations Linked to JAK1/GATA3

Regulatory‐network analysis generated several candidate upstream relationships for the two markers. NetworkAnalyst linked JAK1 to TFs including USF2, YY1, FOXC1, KLF5, and E2F1, while GATA3 was connected with JUN, SP1, NFYA, ELK1, NKX3‐2, FOXL1, and E2F1 (Figure 8A). E2F1 therefore appeared in both TF networks, making it a shared predicted regulator in this analysis. miRNet supplied a broader set of putative post‐transcriptional partners for JAK1 and/or GATA3, among them hsa‐miR‐33b‐5p, hsa‐miR‐34a‐5p, hsa‐miR‐378c, hsa‐miR‐454‐3p, hsa‐miR‐503‐5p, hsa‐miR‐7‐5p, hsa‐miR‐15b‐5p, hsa‐miR‐181b‐5p, hsa‐miR‐210‐3p, and hsa‐miR‐218‐5p (Figure 8B). Drug–gene enrichment analysis based on DSigDB generated several candidate compounds associated with JAK1 and/or GATA3. The five highest‐ranked entries included N’‐Nitrosonornicotine, Vitinoin, trimethyl‐beta‐cyclodextrin, methylprednisolone, and thiostrepton (Table 2), while the complete prediction results are provided in Supporting Information S2: Table S4. These compounds should be regarded as computationally prioritized candidates rather than experimentally validated therapeutic agents. The optimal direction of therapeutic modulation remains uncertain, and additional experimental studies and clinical evaluations are needed before any translational inference can be made.

**Figure 8:**
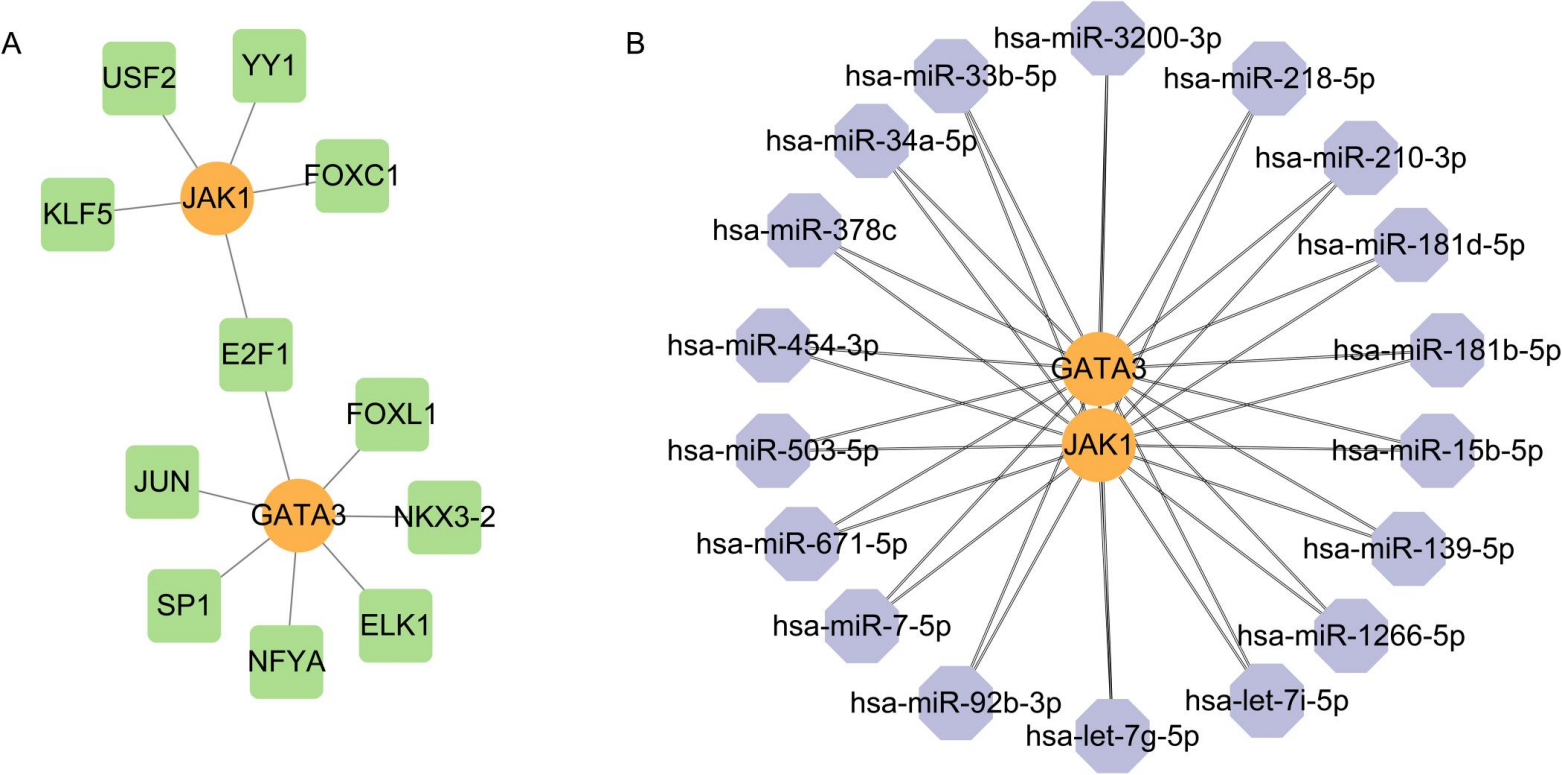
Predicted regulatory networks of JAK1 and GATA3. (A) NetworkAnalyst TF‐gene relationships; the two diagnostic genes are shown in orange and candidate transcription factors in green. (B) miRNet miRNA‐gene relationships, with JAK1/GATA3 in orange and predicted miRNA partners in purple.

**Table 2:** Predicted candidate drugs for the diagnostic genes.

Term	*p*‐value	Adjusted P‐value	Odds Ratio	Combined Score	Genes
N’‐Nitrosonornicotine CTD 00000423	1.30E–03	0.039196703	1665.5	11068.4336	GATA3
Vitinoin CTD 00007069	1.52E–03	0.039196703	38440	249461.7701	GATA3; JAK1
Trimethyl‐beta‐cyclodextrin CTD 00003512	1.90E–03	0.039196703	1110	6955.671007	JAK1
methylprednisolone CTD 00006322	2.00E‐03	0.039196703	1051.526316	6535.342984	JAK1
thiostrepton MCF7 DOWN	0.002198806	0.039196703	951.2857143	5821.7173	GATA3

### 3.9 Identification of Molecular Subtypes Based on JAK1 and GATA3

Consensus clustering of the septic training samples, using only JAK1 and GATA3 expression, favored a two‐group solution. The consensus matrix, cumulative distribution function (CDF) trajectories, and delta‐area profile all supported *k* = 2 (Figure 9A–C). PCA placed the two groups in largely separate regions of the reduced‐dimensional space (Figure 9D). Both markers were higher in Subtype 1 than in Subtype 2 (Figure 9E), and the volcano plot showed a broader transcriptomic difference between the groups (Figure 9F). Genes with higher expression in Subtype1 mapped mainly to immune programs, including Th17 differentiation, Th1/Th2 differentiation, T‐cell receptor signaling, T‐cell differentiation, and immune‐response‐regulating cell‐surface receptor signaling (Figure 9G). Subtype2‐upregulated genes instead favored vesicle/granule annotations such as vesicle lumen, tertiary granule membrane, specific granule lumen, secretory granule membrane, and cytoplasmic vesicle lumen (Figure 9H). The immune profiles followed the same separation. Subtype1 showed higher ssGSEA scores for several adaptive immune populations, including B cells, CD8 + T cells, NK cells, T‐helper cells, TIL‐like signatures, Th1 cells, Th2 cells, and Tregs, along with stronger APC co‐stimulation, cytolytic activity, HLA, T‐cell co‐stimulation, and type I interferon‐response scores (Figure 9I). CIBERSORT identified differences in CD8 + T cells, naive CD4 + T cells, gamma‐delta T cells, and M0 macrophages (Figure 9J), while MCP‐counter showed greater T‐cell, CD8 + T‐cell, cytotoxic‐lymphocyte, and NK‐cell abundance in Subtype1 (Figure 9K). Thus, the two‐gene partition captured one immune‐related axis of sepsis heterogeneity: Subtype1 was JAK1/GATA3‐high and more adaptive‐immune active, whereas Subtype2 displayed weaker adaptive signatures and a vesicle/granule‐oriented transcriptional pattern.

Figure 9JAK1/GATA3‐defined sepsis subgroups and their biological profiles. (A) Consensus matrix plot for *k* = 2. (B) Consensus CDF curves across candidate k values. (C) Delta‐area change used to compare clustering solutions. (D) PCA projection of Subtype1 and Subtype2. (E) Expression distributions of JAK1 and GATA3 between Subtype1 and Subtype2. (F) Subtype differential‐expression volcano plot. (G,H) Representative enrichment results for genes higher in Subtype1 and Subtype2, respectively. (I) ssGSEA comparison of cell signatures and immune functions. (J) CIBERSORT leukocyte composition by subtype. (K) MCP‐counter estimates of immune and stromal abundance.

**Figure:**
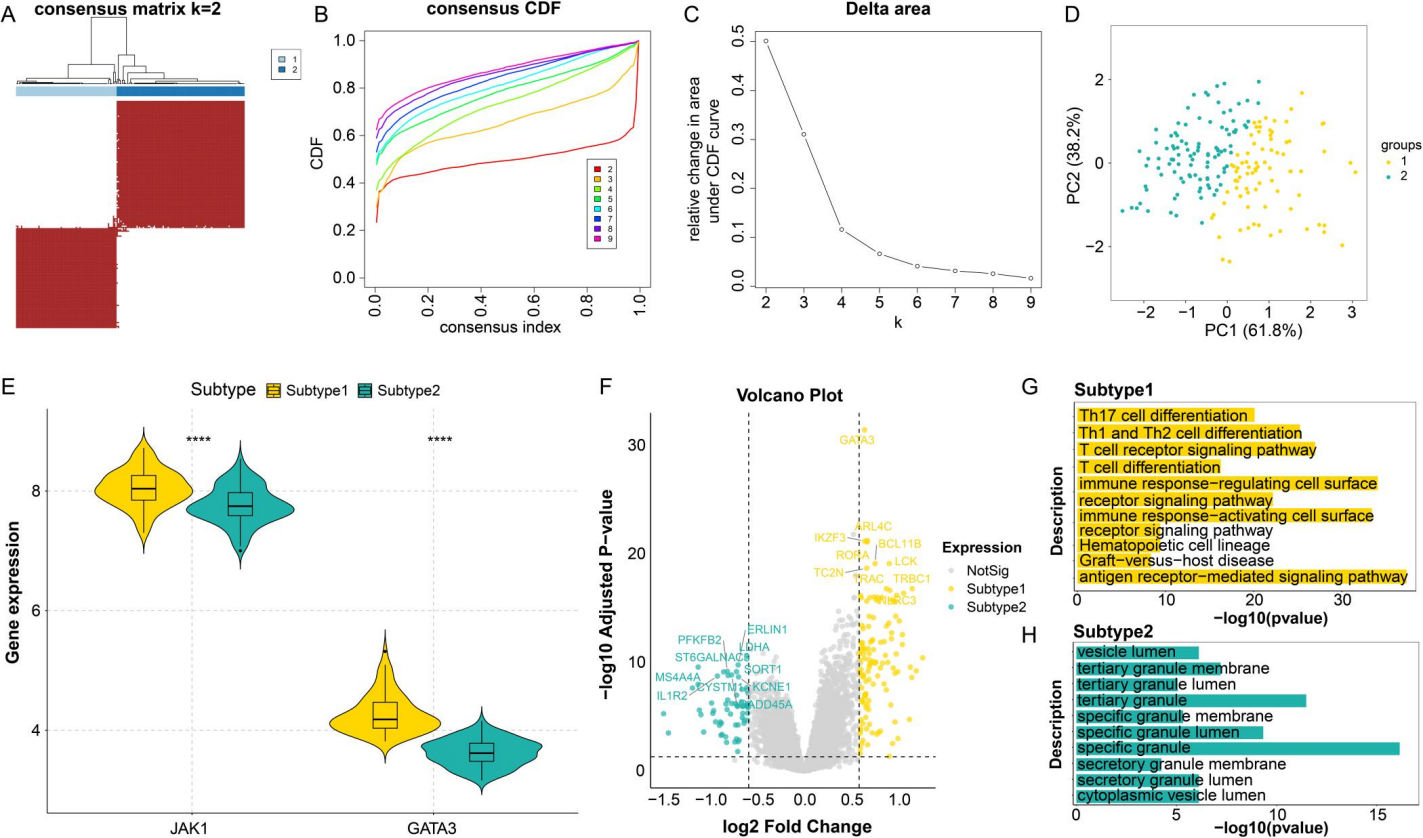

**Figure:**
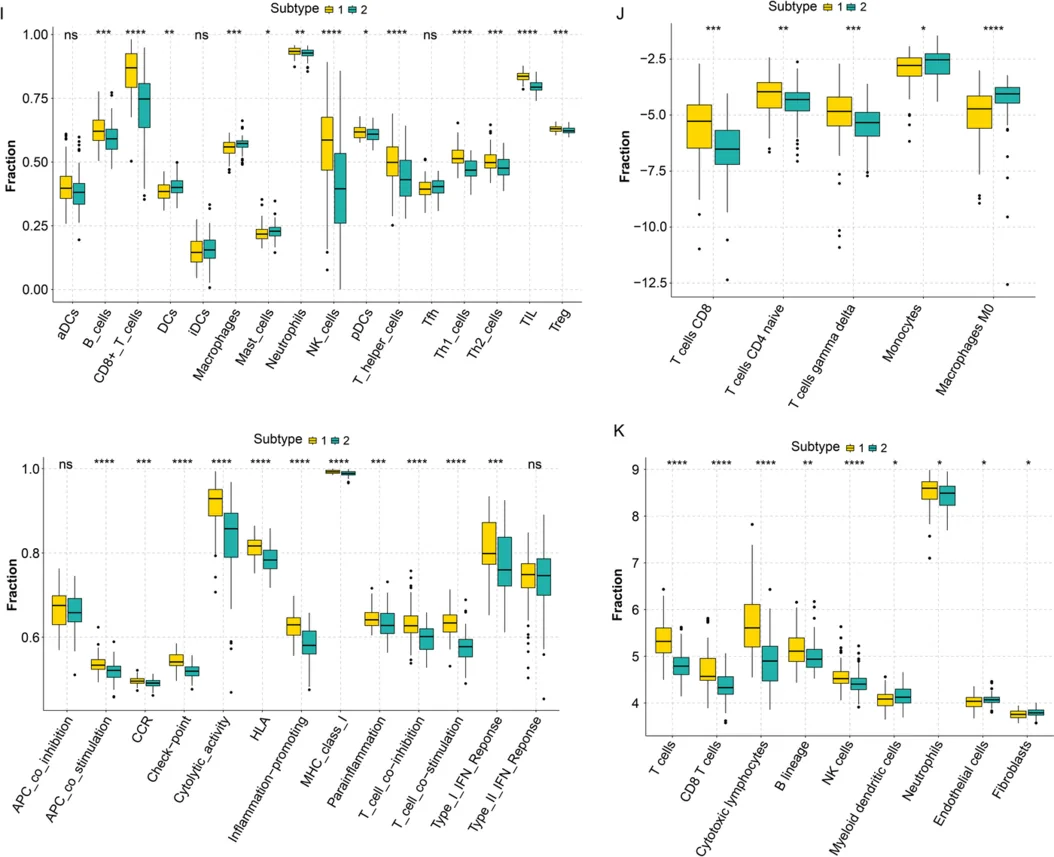

### 3.10 RT‐qPCR Validation of JAK1 and GATA3 Expression

To experimentally examine the expression patterns of the two diagnostic genes, JAK1 and GATA3 were measured in an LPS‐treated HUVECs model by RT‐qPCR. Compared with control cells, LPS‐treated HUVECs markedly reduced the mRNA levels of both JAK1 and GATA3 (both *p* < 0.0001; Figure 10). Notably, the direction of these expression changes was consistent with that observed in the transcriptomic training and external validation cohorts, in which both genes were downregulated in sepsis. These findings provide additional experimental support for the reproducibility of the JAK1/GATA3 expression signature under inflammatory stimulation.

**Figure 10:**
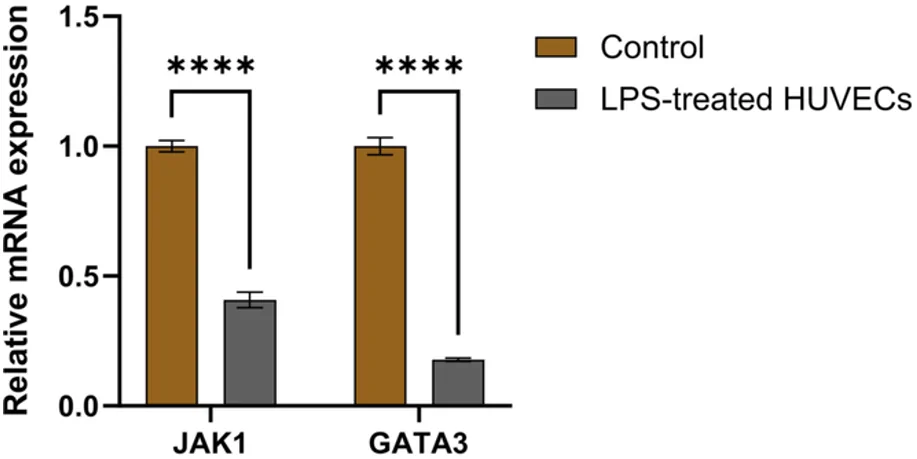
JAK1 and GATA3 transcripts in the LPS‐treated HUVECs model. Values were expressed relative to GAPDH. Control: HUVECs cultured under standard conditions. LPS‐treated HUVECs: Cells treated with 300 ng/mL LPS for 24 h to induce an inflammatory response. Statistical significance was determined by unpaired *t*‐test (*p* < 0.0001). Note that this model represents endothelial inflammation rather than the full spectrum of sepsis.

## 4 Discussion

Our results place JAK1 and GATA3 at the intersection of a GH‐associated transcriptional framework, sepsis discrimination, and immune‐state variation. Both genes were repeatedly selected by the analytical pipeline, were lower in sepsis across independent datasets, and reproduced this direction in LPS‐treated HUVECs. The immune analyses add an important qualification to their diagnostic role: low expression occurred together with weaker lymphocyte and T‐cell signals and a relative increase in myeloid/neutrophil‐associated features, whereas higher expression marked a more adaptive‐immune‐active profile. The two markers therefore appear to report one aspect of host‐response heterogeneity rather than functioning only as binary disease classifiers.

The first biological issue is how the “growth hormone‐related” origin of JAK1 and GATA3 should be interpreted. The present study defined GHRGs through a GeneCards search for “Growth hormone” using a relevance‐score threshold, rather than by restricting the analysis to experimentally established components of the canonical GHR pathway. This distinction is important because the classical GH receptor primarily signals through JAK2, followed by STAT and other downstream pathways [12, 13]. JAK1 should therefore not be described as the canonical kinase directly mediating GH receptor signaling. Instead, JAK1 is better interpreted as part of a broader GH‐related molecular context in which endocrine adaptation intersects with cytokine and immune signaling. This distinction is particularly relevant in sepsis, where inflammatory stress can induce GH resistance and alter JAK/STAT‐associated signaling without necessarily reflecting a primary defect in canonical GH receptor signaling [14]. The broader annotation increased the chance of identifying endocrine‐immune signals, but it also limits how specifically the results can be attributed to direct GH signaling. Nevertheless, previous studies have reported that GH can upregulate GATA3 expression via STAT5B activation, which supports the possibility that GATA3 may be embedded in a broader GH‐related regulatory network, even though this does not imply direct JAK1‐mediated canonical GH receptor signaling [15].

JAK1 is biologically plausible in this wider context because many cytokine receptors depend on it, and interferon signaling is especially sensitive to JAK1 loss. Experimental deficiency of JAK1 markedly impairs responses to both IFN‐alpha/beta and IFN‐gamma [16]. Sepsis‐associated immune dysfunction also involves reduced antigen presentation, altered cytokine responsiveness, and loss of adaptive immune competence [17]. The lower JAK1 expression observed here could therefore mark a state in which several of these programs are attenuated simultaneously. It would be premature, however, to interpret this as direct JAK1‐driven immunosuppression. Whole‐blood expression reflects both regulation within cells and shifts in the proportions of circulating leukocyte populations; altered cytokine exposure may contribute as well. Cell‐type‐resolved experiments will be needed to distinguish among these explanations.

The enrichment analysis suggests that the JAK1‐associated signal may also contain a metabolic component. Sepsis causes extensive metabolic reprogramming in immune and parenchymal cells, and the ability of immune cells to survive and execute effector functions depends heavily on their metabolic state [18]. In our ranked analyses, JAK1 covaried with mitochondrial electron‐transfer and bioenergetic programs in addition to immune pathways. That association is noteworthy because sustained lymphocyte activation requires adequate mitochondrial function and energy supply, whereas metabolic failure can reinforce exhaustion or loss of effector capacity [19]. The current data cannot establish whether JAK1 regulates these mitochondrial programs, whether the same cell populations account for both signals, or whether both simply track disease severity. The result is therefore best treated as a lead for cell‐resolved metabolic and signaling studies rather than as a defined mechanism.

GATA3 provides a complementary view that is more directly connected to adaptive lymphocyte biology. Beyond its well‐known role in Th2 differentiation, GATA3 is required for the maintenance and expansion of mature T cells after antigen‐receptor and cytokine stimulation [20]. A reduction in whole‐blood GATA3 could therefore be consistent with impaired maintenance of adaptive immune activity, particularly because our enrichment results also linked GATA3 to T‐cell signaling and biosynthetic programs needed for active lymphocytes. Independent clinical observations point in the same direction: septic patients have been reported to show lower GATA3 than healthy controls, with persistent suppression among nonsurvivors and a relationship with HLA‐DRA expression [21]. A separate transcriptomic study also reported that T‐cell genes, including GATA3, were inversely associated with organ‐failure burden and mortality, whereas neutrophil‐associated genes correlated positively with disease severity [22]. Together with the present findings, these observations support GATA3 as a candidate indicator of preserved adaptive immune activity in sepsis. However, reduced expression should not be interpreted as evidence that GATA3 itself protects against organ failure or mortality, because changes in circulating T‐cell abundance and activation state may contribute substantially to the measured transcript level.

The immune‐deconvolution analyses provide the strongest internal support for a shared immunological interpretation of JAK1 and GATA3. Although the three computational approaches estimate immune composition differently, they converged on a broadly similar shift from lymphoid‐associated activity toward stronger myeloid and neutrophil‐related signals in sepsis. Such a pattern is compatible with contemporary models of sepsis in which innate inflammatory activity can coexist with lymphocyte depletion, impaired antigen presentation, T‐cell dysfunction, and other forms of adaptive immune suppression rather than progressing through simple, mutually exclusive “hyperinflammatory” and “immunosuppressive” phases [17]. JAK1 and GATA3 were positively related to many lymphocyte‐associated measurements and negatively related to macrophage signals, placing both genes along this broader immune redistribution. In practical terms, low JAK1/GATA3 expression appears to mark an adaptive‐immune‐attenuated, myeloid/neutrophil‐dominant transcriptomic state [23]. Because bulk blood conflates cellular abundance with per‐cell expression, the present data do not show that either gene causes the shift in leukocyte composition.

The unsupervised clustering analysis tested the same relationship from another angle. Septic samples with higher JAK1/GATA3 expression again showed stronger adaptive immune signals, whereas the lower expression subgroup displayed weaker lymphocyte activity and a different vesicle/granule‐associated transcriptomic program. Reappearance of the same immune contrast after clustering supports the idea that the two genes capture a real dimension of host–response variability. It should not, however, be interpreted as a new universal sepsis taxonomy. The classes were generated from only two variables and are more appropriately described as JAK1/GATA3‐defined expression subgroups. Published sepsis endotypes generally rely on much broader signatures and independent replication [24]. Testing stability across infection sources, illness severities, sampling times, and treatment exposures is therefore essential before the subgroups can be considered clinically generalizable.

The diagnostic results are encouraging mainly because the direction of expression and the discriminatory signal persisted outside the discovery datasets. JAK1 and GATA3 were lower in sepsis in both validation cohorts, and the combined model retained useful separation. This cross‐cohort consistency is more informative than the near‐perfect performance observed in the training cohort alone, because it suggests that the signal is not entirely dependent on a single dataset or platform. This observation is consistent with previous work showing that multicohort host‐response transcriptomic signatures can retain diagnostic information across heterogeneous sepsis datasets [25]. At the same time, the exceptionally high AUCs require restraint in claims of clinical readiness. The datasets are retrospective case–control transcriptomic cohorts, and GSE28750 contains only 30 samples. Performance obtained under these conditions may overestimate what can be achieved in prospective clinical populations, where sepsis must be distinguished from heterogeneous inflammatory and infectious mimics rather than from predefined controls. The next critical question is therefore whether JAK1/GATA3 provide incremental diagnostic information in patients with suspected sepsis after accounting for routine clinical variables and established biomarkers. At present, the signature should be regarded as an externally reproducible transcriptomic classifier rather than a clinically validated diagnostic test.

Our integrative approach aligns with a growing body of studies that combine machine learning, network‐based target discovery, single‐cell analysis, and experimental validation to prioritize biomarkers and therapeutic targets. A recent study integrated multiple machine learning models with single‐cell sequencing to identify key target genes of Calculus Bovis in the treatment of sepsis, and further confirmed stable interactions between its active components and target proteins through molecular docking and dynamics simulations [26]. Meanwhile, single‐cell analysis of shared immune regulation in sepsis and systemic lupus erythematosus highlighted the importance of resolving context‐dependent immune heterogeneity and cell‐type‐specific regulatory programs, suggesting that the roles of prioritized candidate genes should be interpreted within specific immune cellular contexts [27]. Although our study did not incorporate single‐cell sequencing, these studies reinforce the importance of interpreting bulk transcriptomic and deconvolution‐based findings in light of cellular composition and cell‐state specificity. Furthermore, integrative bioinformatics and experimental studies in other disease contexts, such as the mechanistic investigation of triphenyl phosphate promoting malignant phenotypes of bladder and kidney cancer via MMP9, underscore the necessity of progressing from computational target nomination to orthogonal functional validation [28]. Compared with these studies, the strength of our work lies in combining a GHRG‐focused computational framework with a two‐gene diagnostic model, immune deconvolution analysis, DSigDB‐based compound inference, and RT‐qPCR validation in an endothelial inflammatory model. At the same time, these comparisons also clarify the boundaries of our current evidence, particularly the need for single‐cell resolution, immune‐cell‐specific validation, and prospective clinical confirmation.

The therapeutic implications must be framed even more cautiously. The finding that JAK1 is reduced in sepsis does not provide a rationale for nonspecific JAK1 inhibition; given the established requirement for JAK1 in interferon and cytokine signaling, further suppressing this pathway could theoretically aggravate some forms of immune dysfunction [16]. Similarly, GATA3 should not be treated as a simple drug target on the basis of a blood‐expression association. A more informative translational question is whether the low‐expression state identifies a subgroup with reversible defects in adaptive immune function and, if so, which cellular pathways could be restored safely without amplifying harmful inflammation. The regulatory‐network and DSigDB analyses in the manuscript should therefore remain explicitly hypothesis‐generating. These database‐derived associations indicate molecular connectivity only and provide no evidence of treatment efficacy, optimal direction of intervention, or safety in sepsis. This caution is especially important for the GH‐related framing: the current data do not justify GH administration, and previous randomized trials found increased morbidity and mortality with high‐dose GH in prolonged critical illness [29].

These translational considerations also highlight the boundaries of the current evidence. The findings were derived from retrospective public microarray cohorts, in which clinical heterogeneity and potential confounding factors could not be fully controlled, making prospective multicenter validation necessary. In addition, bulk transcriptomic profiling and computational immune deconvolution cannot distinguish cell‐intrinsic regulation from changes in leukocyte composition or establish causal roles for JAK1 and GATA3. Although the broad GeneCards‐derived GHRG set was useful for capturing a wider range of endocrine–immune‐associated molecular signals, this breadth also limits pathway specificity because some included genes may be indirectly associated with GH biology rather than representing experimentally established components of canonical GHR signaling. Moreover, the experimental validation of JAK1 and GATA3 was limited to LPS‐stimulated HUVECs, which model endothelial inflammation rather than the full sepsis syndrome and do not directly confirm their proposed roles in T‐cell–mediated adaptive immunity. Validation in PBMCs or other relevant immune‐cell models would be needed to more directly support the inferred immune mechanisms. Finally, both the two‐gene diagnostic model and the JAK1/GATA3‐defined patient groups require confirmation in clinically representative cohorts before their diagnostic or stratification value can be established.

## 5 Conclusion

JAK1 and GATA3 were reproducibly associated with sepsis across the analyzed transcriptomic cohorts and emerged from the GH‐related gene framework as a compact two‐gene diagnostic signature. Lower expression of both markers tracked an immune state with weaker adaptive‐immune features and relatively stronger myeloid/neutrophil signals, indicating that the signature may carry information about host‐response heterogeneity in addition to case–control discrimination. The findings do not demonstrate failure of canonical GH signaling and should remain framed as transcriptomic associations. Validation in prospective clinical cohorts, together with cell‐resolved functional work, will be needed before clinical translation.

## Author Contributions

**Xiuhua Li:** conceptualization, methodology, data curation, formal analysis, validation, writing – original draft. **Ang Liu:** conceptualization, investigation, data curation, visualization, writing – review and editing. **Yingwei Ding:** investigation, software, methodology, Visualization. **Gang Chen:** resources, formal analysis, project administration, software. **Bingwen Zhang:** validation, writing – review and editing, project administration, supervision, resources.

## Funding

The authors have nothing to report.

## Conflicts of Interest

The authors declare no conflicts of interest.

## Supporting information


**Figure S1:** Pairwise sample correlation analysis in GSE57065 and GSE95233.
**Figure S2:** Machine learning‐based validation of candidate diagnostic genes with patient‐level ten‐fold cross‐validation.
**Figure S3:** Machine learning‐based validation of candidate diagnostic genes from the MsigDB and literature‐derived GHRG datasets.
**Figure S4:** Clinical utility assessment of the two‐gene model in the GSE26378 cohort.

**Table S1:** Detailed information of the included GEO datasets.
**Table S2:** List of 1,205 growth hormone‐related genes retrieved from the GeneCards database.
**Table S3:** Machine Learning results.
**Table S4:** Drug prediction results(DSigDB).

## Data Availability

All data analyzed in this work were downloaded from public databases. Relevant database accession information has been provided in the manuscript.
