# Supplementary material for: Growth Hormone‐Related Gene Signatures Reveal Diagnostic Biomarkers, Immune Landscapes, and Potential Therapeutic Targets in Sepsis Through WGCNA and Machine Learning Analyses
Source: Immun Inflamm Dis. 2026 Oct 28;14(10):e70566. doi: 10.1002/iid3.70566 (PMC13715502; doi:10.1002/iid3.70566)
Supplement: Supplementary file 1 [file IID3-14-e70566-s002.docx]

**Supplemental Figure**

Supplemental Figure S1. Pairwise sample correlation analysis in GSE57065 and GSE95233. (A) Correlation heatmap for GSE57065 using the top 2,000 HVGs. (B) Correlation heatmap for GSE95233 using the top 2,000 HVGs. Note that repeated samples from the same patient (indicated by color bars) do not always exhibit the highest similarity.

Supplemental Figure S2. Machine learning-based validation of candidate diagnostic genes with patient-level ten-fold cross-validation. (A) LASSO coefficient trajectories as regularization changes. (B) Cross-validation profile used to choose the LASSO penalty. (C) XGBoost gain-based feature ranking. (D) SVM-RFE cross-validation error across candidate feature-set sizes. (E) Random-forest error as tree number increased. (F) Random-forest importance metrics. (G) UpSet plot showing the intersection of the four selected gene lists.

Supplemental Figure S3. Machine learning-based validation of candidate diagnostic genes from the MsigDB and literature-derived GHRG datasets. (A) Three-way overlap of DEGs, WGCNA-derived genes, and GHRG genes obtained from MsigDB and the literature. (B) LASSO coefficient trajectories as regularization changes. (C) Cross-validation profile used to choose the LASSO penalty. (D) XGBoost gain-based feature ranking. (E) SVM-RFE cross-validation error across candidate feature-set sizes. (F) Random-forest error as tree number increased. (G) Random-forest importance metrics. (H) UpSet plot showing the intersection of the four selected gene lists.

Supplemental Figure S4. Clinical utility assessment of the two-gene model in the GSE26378 cohort. (A) Decision curve for the paired-gene model. (B) Calibration of predicted sepsis probability against the observed outcome.
